# From fat to facts: Anthropometric references and centile curves for sum of skinfolds and waist-to-hip ratio in 2,507 adults

**DOI:** 10.1371/journal.pone.0326111

**Published:** 2025-06-26

**Authors:** Francesco Campa, Giuseppe Coratella, Cristian Petri, Fabrizio Spataro, Davide Charrier, Giuseppe Cerullo, Giulia Baroncini, Eleonora Faraone, Giorgio Pio Alberto Marinelli, Sofia Serafini, Salvatore Vaccaro, Matteo Pincella, Pascal Izzicupo, Antonio Paoli

**Affiliations:** 1 Department of Biomedical Sciences, University of Padua, Padua, Italy; 2 Department of Biomedical Sciences for Health, Università degli Studi di Milano, Milano, Italy; 3 Department of Sports and Computer Science, Section of Physical Education and Sports, Universidad Pablo de Olavide, Seville, Spain; 4 Section of Clinical Nutrition and Nutrigenomics, Department of Biomedicine and Prevention, University of Rome Tor Vergata, Rome, Italy; 5 Milan Lab Department, AC Milan, Milan, Italy; 6 Department of Science and Technology for Humans and the Environment, Università Campus Bio-Medico di Roma, Rome, Italy; 7 Department of Neuroradiology, ASST Spedali Civili di Brescia, Brescia, Italy; 8 Department of Medicine and Aging Sciences, “G. D’Annunzio” University of Chieti-Pescara, Chieti, Italy; 9 Clinical Nutrition Unit and Oncological Metabolic Centre - Endocrinology Department, Azienda Unità Sanitaria Locale – IRCCS di Reggio Emilia, Reggio Emilia, Italy; 10 FIGC Federazione Italiana Giuoco Calcio (Italian Football Federation), Rome, Italy; University of Verona: Universita degli Studi di Verona, ITALY

## Abstract

**Background and aims:**

Direct assessment of skinfold thickness and waist and hip girths provides information about body fat and its distribution, avoiding estimation errors due to predictive equations. The present study aimed to provide new centile curves for the sum of eight skinfold thicknesses (Σ8SKF) and waist-to-hip ratio (WHR) in adult population, and to identify breakpoints during adulthood.

**Methods:**

The present investigation was conceived as a multicenter, cross-sectional study. Stature, body mass, eight skinfold thicknesses (i.e., triceps, biceps, subscapular, iliac crest, supraspinal, abdominal, thigh, and calf) and waist and hip girths were measured according to the International Society for the Advancement of Kinanthropometry protocol in 1,313 men and 1,194 women aged 18–65 years. Smoothed age- and sex-specific percentile curves were generated using the Lambda Mu and Sigma method. For both sexes, simple linear regressions of the dependent variable (Σ8SKF and WHR) versus the explanatory variable (age) were performed to investigate changes in the response variable’s slope and to test for potential breakpoints.

**Results:**

Reference percentile curves (3rd, 10th, 25th, 50th, 75th, 90th, and 97th) for Σ8SKF and WHR were provided. In men, Σ8SKF increased by 1.0 mm/year between the ages of 21 and 59, while in women, it increased by 3.8 mm/year between the ages of 38.5 and 47. In men, WHR showed a progressive increase of 0.004/year until the age of 28.4, followed by a slower increase of 0.003/year throughout the lifespan. In women, WHR increased by 0.003/year from the age of 20–65.

**Conclusions:**

Σ8SKF and WHR appear sex- and age-specific. Scientists and practitioners are provided with reference values for the adult population.

## Introduction

In evaluating health status, body mass linked to stature is one of the main aspects that can be monitored for lifespan and is expressed as body mass index (BMI), allowing for classification into under-, normal-, over-weight and obese people [1]. Based only on mass and stature, BMI is easy and fast to administer and has been used worldwide [1]. However, unlike stature, body mass depends on the magnitude of many components whose sum can be constant even though they may vary over time, having possibly an impact on the health status [2]. Therefore, rather than the gross body mass, determining body composition may reflect better the people’s health status. In this regard, body fat can be used to monitor various diseases such as metabolic and cardiovascular when in excess, and hormonal and immune deficits when scarce [3,4]. The quantification of body fat can be made using different procedures [5], and the most accurate methods require scanning protocols using magnetic resonance or computed tomography imaging, albeit these necessitate costly devices mostly not available for practitioners. Other laboratory techniques include dual-energy X-ray absorptiometry, ultrasonography, hydrostatic weighing, and air displacement plethysmography with the same limitations as above. For these reasons, alternative techniques such as bioelectrical impedance analysis and anthropometric-based procedures have been developed over the years [6,7].

When anthropometric techniques are used to assess body composition in order to improve health or performance, this translates into kinanthropometry, a discipline that is currently receiving widespread interest and application [6]. The quantification of body fat through anthropometric-based procedures derives mainly from the skinfold thickness measured in different body sites, inserted in predictive equations [6]. Although easier and cheaper than the laboratory techniques mentioned above, the measurement of the skinfold thickness requires accurate and reliable protocols defined by international guidelines [8]. Importantly, multiple predictive equations have been developed depending on factors like sex, age, and ancestry and are specific for athletes and non-athletes [6]. Consequently, this implies that the predictive equation for a given person should be chosen considering all factors together, and practitioners may not be aware of the existence of all the equations. Additionally, each equation has been developed using a specific population, and not being in line with the exact characteristics of such a population may result in a systematic error.

To facilitate the process, one may consider summing the skinfolds without the need to insert them into predictive equations, thus freeing the results from any error deriving from the mathematical process [9–11]. The international guidelines report that the skinfold of the triceps, biceps, subscapular, iliac crest, supraspinal, abdominal, thigh, and calf are the most representative of the human fat distribution [8]. The sum of these eight skinfolds (Σ8SKF) has shown a correlation with the amount of total body fat [12–14], making the use of equations not strictly necessary. However, while the outcomes of predictive equations can be easily interpreted as body fat to body mass, i.e., percentage, [6], the Σ8SKF has no reference in the literature for adults while available only for athletes [9,10]. It is worth noting that values for the athletic population, particularly endurance athletes and team sport athletes, are generally lower compared to the general population of similar age [9,10]. However, there is no comprehensive lifespan reference available for Σ8SKF in the current literature.

The body fat distribution can also be assessed using the waist-to-hip ratio (WHR), i.e., the ratio between the waist and hip girths [15]. WHR has been strongly advocated to be used to redefine obesity together with other parameters [15]. However, while the literature has provided references for young people [16,17], no representative data are available for adults and no lifespan analysis is available. Therefore, the present study aimed to: i) provide centile references for Σ8SKF and WHR in adults and ii) provide lifespan analysis to estimate how both change over time.

## Materials and methods

All research procedures were reviewed and approved by the Ethical Committee board of the University of Padova (approval code: HEC-DSB/02–2023) and are conform to the Declaration of Helsinki concerning studies involving human subjects. After being provided with a detailed written explanation of the procedures, the participants gave their written informed consent.

### Participants

Recruitment took place through advertisements placed in universities, medical, recreational, market, and sports centers across Italy, running from 01/01/2024–01/01/2025. We used a stratified sampling method based on age categories, within which individuals were randomly selected to ensure accurate representation. For each age group, a minimum number of subjects was recruited to ensure a normal distribution of the data, in accordance with previous studies [18,19]. The selection was also based on ensuring that at least 90% of the participants displayed a similar ethnicity (Caucasian). Exclusion criteria included the inability to collect all selected anthropometric measurements, pregnancy, or being outside the age range of 18–65 years. A total of 2,507 participants aged from 18 to 65 years, 1,313 men and 1,194 women were involved in this study.

### Procedures

The present investigation was conceived as a multicenter, cross-sectional study. The study collected data at a national level from multiple cities across various Italian territories (Milano, Padova, Reggio Emilia, Firenze, Chieti, Pescara, and Roma). The anthropometric assessments were conducted by operators certified by the International Society for the Advancement of Kinanthropometry, following international standards [8]. Body mass and stature were measured using a scale with an integrated stadiometer (Seca, Hamburg, Germany), with a sensitivity of 0.1 kg and 0.1 cm, respectively. BMI was calculated as body mass (kg) divided by squared stature (m²).

Skinfold thicknesses at the triceps, biceps, subscapular, iliac crest, supraspinal, abdominal, thigh, and calf sites were measured using three different calipers (Holtain Ltd, United Kingdom; Harpenden, Baty International Ltd, West Sussex, UK; Cescorf, Porto Alegre, Brazil), each with a sensitivity of ±0.1 mm and a pressure range between 8.0 g/mm² and 10.0 g/mm². To ensure consistency, the interclass correlation coefficient (ICC) was calculated for the three calipers based on a sample of 20 participants, yielding ICC = 0.95 (95% CI: 0.93–0.97). Waist and hip girths were measured using a similar measuring tape (Lufkin, Apex Tool Group, USA) with a sensitivity of ±0.1 mm. The WHR was computed as the ratio of waist girth to hip girth. The reproducibility of Σ8SKF and WHR measurements among the three different operators (F.C., G.C., and D.C.) was assessed. The analysis demonstrated excellent reliability, with an ICC of 0.92 (95% CI: 0.88–0.96) and 0.94 (95% CI: 0.92–0.97) for Σ8SKF and WHR, respectively.

## Statistical analysis

Statistical analysis was conducted using R (version 3.4.1) and Lambda Mu and Sigma (LMS) method (LMS chart-maker Pro version 2.4, 2008). The mean ± standard deviation was calculated for each variable. Normal distribution of data was evaluated using the Shapiro–Wilk test. Smoothed age and sex-specific percentiles (3^rd^, 10^th^, 25^th^, 50^th^, 75^th^, 90^th^, and 97^th^) for Σ8SKF and waist-to-hip ratio were generated. Percentiles for the sum of six skinfolds (triceps, subscapular, supraspinal, abdominal, thigh, and calf skinfolds) and waist-to-height ratio are also calculated. The LMS method was used to graphically provide the annual rate of change of Σ8SKF and WHR, with three reference curves representing the median (M), the coefficient of variation (S), and the power to remove skewness from the data (L) by age and was implemented in the Generalized Additive Model for Location, Scale, and Shape (GAMLSS) package included in R software. In the LMS method, GAMLSS parameters and the parameters of Box–Cox power exponential distribution were used for model fitting to data. These reference curves were fitted to the original data and the best fit was used to construct smoothed percentile curves. After the application of the BoxCox power transformation, the data at each age were normally distributed and the points on each percentile curve were defined in terms of the formula: M = (1 + LSz) 1/L where L, M, and S are values of the fitted curves at each age, and z indicates the z-score for the required percentile. For both sexes, simple linear regressions of the dependent variables (Σ8SKF and WHR) vs. the explanatory variable (age) were empirically investigated and tested for changes in the response variables’ slope (Davies test) and for the existence of time points (Pscore test). To identify the time point(s) where a change in the slope of phase angle is observed, we performed a segmented regression analysis using the “segmented” package (v 1.0.0), selecting the model with the lower Bayesian information criterion value. Delta method and sandwich estimator for the standard errors were used to compute 95% confidence interval (CI) of the time point estimates. The slope coefficient estimates and the related 95% Cis were reported, and significant slopes were detected using p-value set at <0.05.

## Results

**Fig 1:**
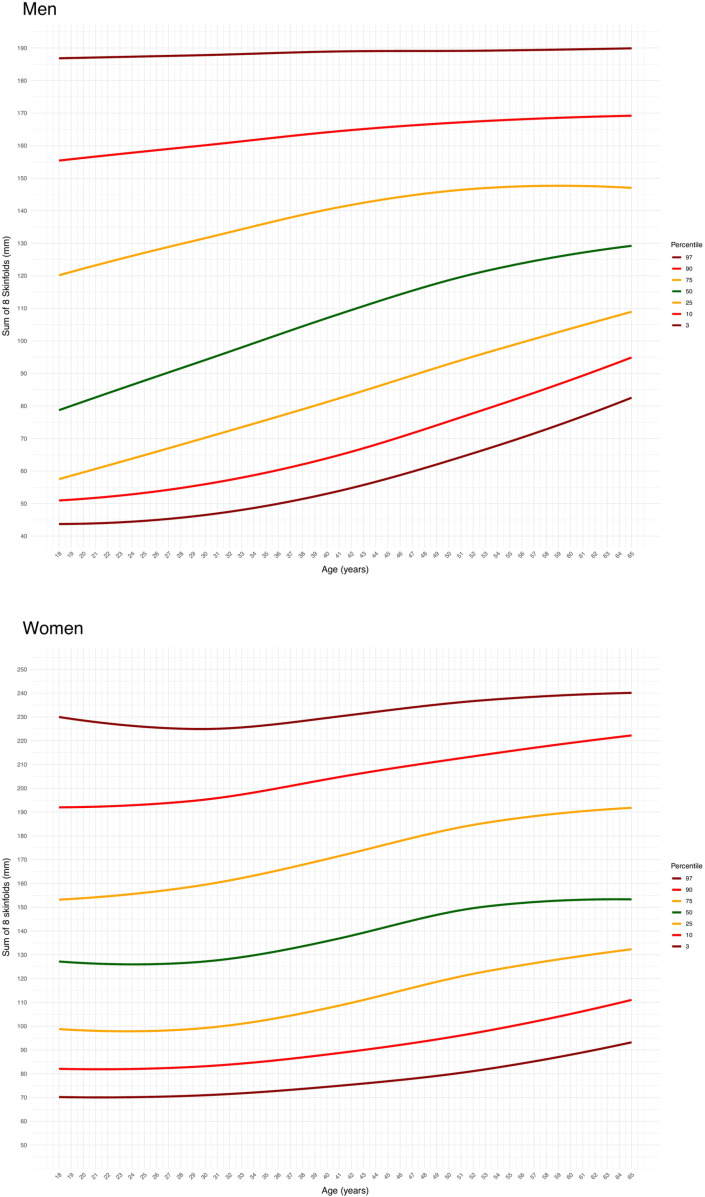
The reference percentile curves for the sum of eight skinfold (Σ8SKF) in male (upper panel) and female (lower panel) participants.

**Fig 2:**
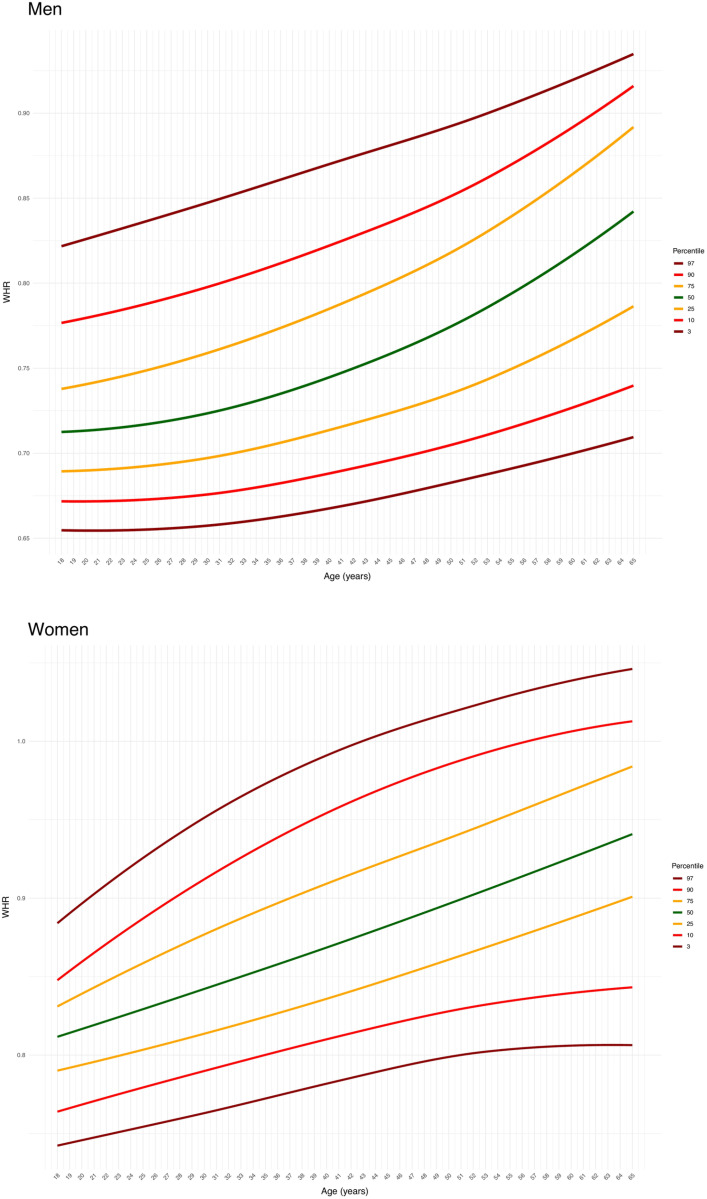
The reference percentile curves for the waist-to-hip ratio (WHR) in male (upper panel) and female (lower panel) participants.

The detailed anthropometric characteristics of the participants are reported in Supplementary S1 and S2 Tables, for men and women, respectively. The reference centile curves for Σ8SKF and WHR are presented in Figs 1 and 2 for men (upper panels) and women (lower panels). The corresponding mean values are detailed in Tables 1 and 2. Reference centile curves for the sum of six skinfolds (triceps, subscapular, supraspinal, abdominal, thigh, and calf skinfolds) and waist-to-height ratio are also provided in Supplementary S3 and S4 Tables, respectively.

**Table 1:** Sum of eight skinfolds (mm) reference percentiles.

Age (years)	3rd		10th		25th		50th		75th		90th		97th	
	Men	Women	Men	Women	Men	Women	Men	Women	Men	Women	Men	Women	Men	Women
<20	47.6	71.5	55.2	81.8	61.8	101.8	89.6	134.2	140.9	163.6	175.6	200.8	200.0	233.4
20-24	42.0	66.8	50.3	80.0	59.0	96.9	82.2	122.2	119.4	157.8	148.0	183.5	183.8	210.5
25-29	43.9	72.5	54.6	84.8	66.6	97.0	84.6	121.0	116.6	151.3	155.0	195.1	181.4	236.2
30-34	50.2	71.9	58.8	85.2	77.1	97.7	98.2	125.9	139.9	156.1	159.8	186.8	183.9	206.5
35-39	49.6	74.1	57.6	84.6	73.9	105.3	98.9	135.8	135.4	169.7	166.7	206.9	192.8	224.1
40-44	55.3	82.9	67.9	99.7	86.7	121.9	116.5	152.5	148.0	197.4	172.2	224.2	199.1	255.5
45-49	58.3	81.5	69.5	99.1	83.5	138.2	110.3	161.1	140.2	187.0	161.0	215.8	189.1	236.3
50-54	61.8	75.9	74.7	96.0	91.6	126.8	122.4	158.6	146.6	189.6	163.1	219.1	179.6	249.9
55-59	84.1	70.3	98.5	84.2	114.6	97.7	131.8	126.3	150.1	150.1	170.6	195.5	187.2	239.4
≥60	73.3	95.5	84.9	109.9	101.9	124.3	124.2	144.5	146.2	186.3	169.1	213.8	192.2	229.3

**Table 2:** Waist-to-hip ratio reference percentiles.

Age (years)	3rd		10th		25th		50th		75th		90th		97th	
	Men	Women	Men	Women	Men	Women	Men	Women	Men	Women	Men	Women	Men	Women
<20	0.75	0.65	0.77	0.67	0.80	0.68	0.82	0.71	0.85	0.74	0.87	0.76	0.92	0.79
20-24	0.74	0.66	0.77	0.67	0.81	0.69	0.84	0.72	0.88	0.76	0.91	0.81	0.96	0.87
25-29	0.74	0.65	0.79	0.68	0.83	0.69	0.86	0.72	0.89	0.75	0.93	0.78	0.95	0.84
30-34	0.77	0.67	0.80	0.68	0.83	0.71	0.85	0.74	0.90	0.77	0.94	0.82	0.98	0.88
35-39	0.79	0.67	0.81	0.69	0.83	0.72	0.87	0.76	0.91	0.81	0.98	0.84	1.04	0.88
40-44	0.78	0.68	0.82	0.71	0.85	0.74	0.89	0.76	0.93	0.81	0.98	0.84	1.02	0.88
45-49	0.78	0.69	0.83	0.72	0.87	0.74	0.90	0.78	0.95	0.81	1.01	0.83	1.05	0.89
50-54	0.80	0.70	0.85	0.71	0.89	0.75	0.93	0.79	0.96	0.84	1.00	0.88	1.03	0.91
55-59	0.75	0.67	0.77	0.69	0.79	0.71	0.81	0.73	0.82	0.75	0.85	0.80	0.87	0.86
≥60	0.77	0.70	0.82	0.73	0.88	0.77	0.93	0.83	0.98	0.88	1.02	0.91	1.07	0.93

**Fig 3:**
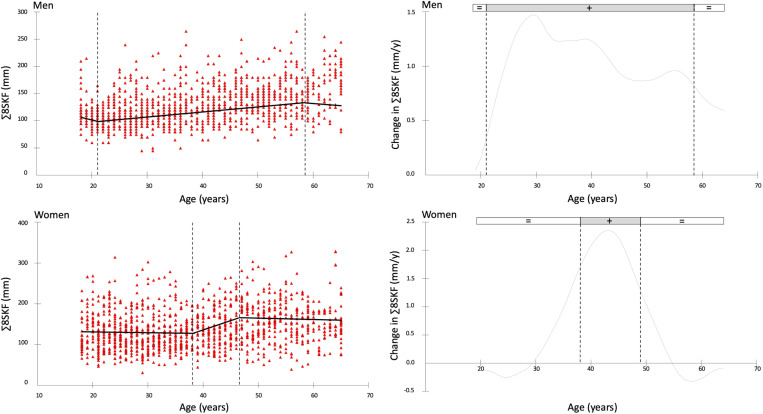
The time points of change for the sum of eight skinfolds (Σ8SKF) are shown on the left, and the annual rate of change on the right, with data for men presented in the upper panels and women in the lower panels.

**Fig 4:**
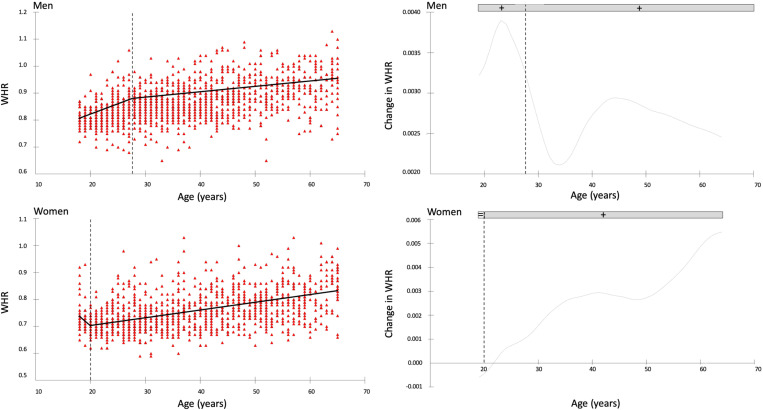
The time points of change for the waist-to-hip ratio (WHR) are shown on the left, and the annual rate of change on the right, with data for men presented in the upper panels and women in the lower panels.

Figs 3 and 4 illustrate the annual rate of change in Σ8SKF and WHR for men (upper panel) and women (lower panel), respectively. The vertical dotted lines indicate the time points at which significant changes occur in the trends. Table 3 presents the time points of change in years and the mean change for each period.

**Table 3:** Sum of eight skinfolds and waist-to-hip ratio trajectories of the participants.

Variable	Breakpoints (years)		Rate of change per year					
	Breakpoint 1	Breakpoint 2	Slope 1 (95% CI)	p-value	Slope 2 (95% CI)	p-value	Slope 3 (95% CI)	p-value
Men								
∑8SKF (mm)	21.0	59.0	−3.9 (−18.1; 10.2)	0.572	1.0 (0.7; 1.3)	<0.001	−1.1 (−5.9; 3.7)	0.636
WHR	28.4	–	0.004 (0.002; 0.006)	<0.001	0.003 (0.001; 0.003)	<0.001	–	–
Women								
∑8SKF (mm)	38.5	47.0	−0.3 (−1.1; 0.5)	0.467	3.8 (0.3; 7.3)	0.035	−0.2 (−1.0; 0.5)	0.517
WHR	20.0	–	−0.02 (−0.06; 0.02)	0.189	0.003 (0.002; 0.003)	<0.001	–	–

Note: CI = confidence interval; ∑8SKF = sum of eight skinfolds; WHR = waist to hip; statistically significance set at p < 0.05.

## Discussion

The present study aimed to provide percentile curves for raw anthropometric measures of Σ8SKF and WHR in adult men and women. A second objective was to describe the rate of change of these parameters over the years. Percentile curves at the 3^rd^, 10^th^, 25^th^, 50^th^, 75^th^, 90^th^, and 97^th^ percentiles were generated, and breakpoints were identified. These references can be used to compare the characteristics of the measured subjects, either by utilizing the provided charts or by referring to the average values for different age and sex categories. For men, Σ8SKF showed a yearly increment of 1 mm from ages 21–59, while for women an increment of 3.8 mm per year was observed between ages 38.5 and 47. Regarding WHR, a progressive increment was recorded in men until the age of 28.4 years, with an annual rate of 0.004, followed by a slower increment of 0.003 per year in the subsequent ages. In women, the increment began at the age of 20, with a rate of 0.003 per year up to 65 years. Such novel data may facilitate the reference of body fat and WHR in adults.

Percentiles for men and women are now available for the first time for Σ8SKF and WHR. The Σ8SKF has been associated with the percentage of body fat measured by reference procedures such as DXA, air displacement plethysmography, underwater weighing, or 4-compartment model [6], and percentiles for body fat have been provided using DXA [19,20]. However, although all reference methods, a lack of agreement was observed between them, making the results procedure- and device-specific [21,22]. Additionally, these reference methods are not easily available for practitioners, thus inducing the search for alternative methods, since only useful for equipped laboratories. Consequently, anthropometric-based procedures have been advocated since based on dimensional data easily collectible to be inserted into validated and specific predictive equations [6]. Nevertheless, these equations are strictly population-specific, and an inappropriate use would result in divergent outcomes [23]. For all the above, categorizing people by body fat has not been possible, as done for BMI since based on direct assessments [24,25]. The present percentiles quantify clearly the body fat in the reference adult population, making possible an individualized diagnosis based on the amount of the subcutaneous adipose tissue and on the fat distribution. This would allow a future categorization of both Σ8SKF and WHR for adults and other populations with respect to health-related risk factors.

In men, a consistent accumulation of subcutaneous adipose tissue is observed from 20 to 59 years of age, followed by a plateau. A similar trend has been reported for total body fat percentage across the male lifespan [19]. Interestingly, total energy expenditure shows a decline until around 20 years of age, stabilizes up to 60, and then decreases further [26]. As for men, this would imply that the total body fat accumulation may derive from other factors, should the total energy expenditure remain similar between 20 and 60 years old. Indeed, a decline in the growth hormone (GH) blood activity, which peaks during childhood and adolescence, begins to occur after the completion of physical growth [27]. In healthy adults, the age-related decline in growth hormone levels is accompanied by a parallel decrease in serum insulin-like growth factor 1 (IGF-1) [28]. The reduction in serum IGF-1 levels suggests a down-regulation of the GH/IGF-1 axis that has been strongly associated with increased fat accumulation and reduced muscle mass [28]. After 60 years of age, while a reduction in energy intake and appetite is reported, the plateau in body fat accumulation may be influenced by hormonal changes, including reduced testosterone and leptin activity, and age-related factors such as decreased sensory perception and physical activity, as well as psychological and health-related condition [29,30].

Women exhibited a narrower window of body fat accumulation ranging from 38.5 to 47 years old, then showing a plateau. Such a period of increment could be a consequence of the perimenopausal phase which begins in the late 30s to early 40s [31,32] and marks the transitional period beginning with the first clinical, biological, and endocrine signs of impending menopause [31,32]. Indeed, during perimenopause women often experience changes in body composition, including decrements in muscle mass and increased body fat [31,32]. In addition, body fat may also change due to psychological aspects, including the influence of various diets and habits related to physical activity, often associated with the changes in hormonal status [33].

WHR is often used to assess the distribution of the body fat accumulation and has been advocated to comprehensively evaluate the risk of metabolic and cardiovascular diseases [15]. Indeed, accumulating fat on the abdominal area is associated with the accumulation of visceral fat that is metabolically active and increases the risk for metabolic disorders, including cardiovascular disease, type 2 diabetes, and metabolic syndrome [15]. Importantly, the World Health Organization has set the WHR cut-off values as more than 1.0 and 0.85 for men and women, respectively, that increase abdominal obesity and reflect substantially an increment in cardiometabolic risks [34]. The present percentiles provide now references for the adult population to be used lifespan.

While body fat distribution differs inherently between men and women, with males generally exhibiting a typical android (abdominal) fat distribution and females showing a typical gynoid (hip) distribution, the WHR tends to increase across the lifespan in both sexes. Aging is often accompanied by a reduced insulin sensitivity that further contributes to storing fat in the abdominal area [35], and consequently around the internal organs [36]. As for men, the increments in WHR may couple with the increments in Σ8SKF, suggesting that body fat progressively accumulates on the abdominal area. In women, the constant increase in WHR suggests a progressive shift in body fat accumulation towards the abdominal area. Interestingly, fat accumulation appears to be more variable and responsive to changes in the abdominal region compared to the lower body [37], which may explain why women tend to progressively store fat also in the abdominal area over time. Indeed, while men and women start with different body fat distributions, they both exhibit a progressive increase in abdominal fat accumulation, which begins slightly before the age of 30 in men and around the early 20s in women.

The present study comes with some acknowledged limitations. Although affordable and user-friendly, the skinfold assessment requires a certain level of expertise that could affect the results should the guidelines not be followed correctly. Moreover, while many calipers are available on the market, some of them are made by low-quality material such as plastic and do not preserve a controlled and constant pressure on the caliper jaws. Additionally, there are other protocols in the literature different from ISAK [6], meaning our results might not be generalizable to other measurement standards. Similarly, as mentioned, our findings may not be directly applicable to measurements taken with calipers that differ significantly in design or technical specifications. Furthermore, our study did not include data correlating skinfold measurements with health outcomes. Identifying cut-off points where skinfold sum values are associated with cardiometabolic parameters, or below which certain pathologies might be detected, could provide valuable insights. This remains an avenue for future research. Lastly, our results apply to the adult population here examined and should not be expanded to other populations with different ancestry or age.

## Conclusions

The present study showed that subcutaneous adipose tissue increments during specific periods in both adult men and women, together with a progressive constant redistribution of body fat towards the abdominal area. It is now possible to refer to age- and sex-specific 3^rd^, 10^th^, 25^th^, 50^th^, 75^th^, 90^th^, and 97^th^ percentiles, considering that time points of increment in Σ8SKF occur from 21 years to 59 years in men, and from 38.5 to 47 years in women. Meanwhile, WHR gradually increments after the age of 28.4 in men and after 20 in women. The new percentile references and the identified time points of change in this study can provide valuable support to researchers and practitioners for monitoring body composition and selecting appropriate nutritional and training strategies aimed at improving it.

## Supporting information

S1 Table Descriptive characteristics of male participants according to age categories. (DOCX)

S2 Table Descriptive characteristics of female participants according to age categories. (DOCX)

S3 Table Sum of six skinfolds (mm) reference percentiles. (DOCX)

S4 Table Waist-to-height ratio reference percentiles. (DOCX)

S5 Dataset Anonymized participant data. (XLSX)

S6 File STROBE checklist. (PDF)
