# Supplementary material for: From fat to facts: Anthropometric references and centile curves for sum of skinfolds and waist-to-hip ratio in 2,507 adults
Source: PLoS One. 2025 Jun 26;20(6):e0326111. doi: 10.1371/journal.pone.0326111 (PMC12200776; doi:10.1371/journal.pone.0326111)
Supplement: S1 Table — (DOCX) [file pone.0326111.s001.docx]

| **Supplementary table 1**. Descriptive characteristics (mean ± standard deviation) of male participants according to age categories. | | | | | | | | | | | | | | |
| --- | --- | --- | --- | --- | --- | --- | --- | --- | --- | --- | --- | --- | --- | --- |
| Age | N. | Body mass (kg) | Height (cm) | BMI (kg/m^2^) | Triceps SKF (mm) | Subscapular SKF (mm) | Biceps SKF (mm) | Iliac crest SKF (mm) | Supraspinal SKF (mm) | Abdominal SKF (mm) | Tight SKF (mm) | Calf SKF (mm) | Waist girth (cm) | Hip girth (cm) |
| <20 | 59 | 79.7 ± 10.8 | 178.3 ± 6.7 | 25.0 ± 2.8 | 11.5 ± 5.6 | 12.4 ± 5.9 | 4.7 ± 3.9 | 15.3 ± 9.8 | 15.1 ± 9.5 | 19.7 ± 10.0 | 16.0 ± 9.7 | 9.5 ± 7.2 | 79.4 ± 6.7 | 98.1 ± 6.9 |
| 20-24 | 182 | 79.0 ± 12.5 | 177.8 ± 7.3 | 24.9 ± 3.2 | 9.6 ± 4.7 | 12.2 ± 5.9 | 4.1 ± 2.0 | 14.3 ± 8.4 | 12.0 ± 7.7 | 17.9 ± 9.9 | 14.3 ± 6.7 | 8.2 ± 5.3 | 80.2 ± 7.6 | 97.6 ± 6.9 |
| 25-29 | 179 | 77.0 ± 10.9 | 176.9 ± 7.3 | 24.6 ± 2.8 | 10.2 ± 5.0 | 12.4 ± 6.1 | 4.4 ± 2.7 | 13.9 ± 7.7 | 13.6 ± 7.7 | 18.9 ± 9.9 | 14.3 ± 7.0 | 8.2 ± 5.3 | 81.2 ± 8.4 | 96.5 ± 6.4 |
| 30-34 | 181 | 79.4 ± 11.5 | 176.9 ± 7.1 | 25.3 ± 3.1 | 10.6 ± 5.2 | 14.5 ± 6.9 | 4.1 ± 1.7 | 15.9 ± 8.4 | 15.6 ± 8.4 | 22.9 ± 10.1 | 15.3 ± 7.5 | 8.4 ± 5.0 | 83.1 ± 7.8 | 97.1 ± 6.2 |
| 35-39 | 172 | 79.4 ± 10.8 | 177.5 ± 6.9 | 25.2 ± 3.0 | 10.8 ± 5.2 | 14.3 ± 7.1 | 4.5 ± 2.8 | 15.4 ± 7.9 | 14.9 ± 8.3 | 23.2 ± 10.5 | 15.3 ± 6.9 | 8.6 ± 5.2 | 83.9 ± 8.3 | 97.4 ± 6.1 |
| 40-44 | 152 | 82.3 ± 12.2 | 177.7 ± 6.5 | 26.0 ± 3.3 | 11.4 ± 4.6 | 16.2 ± 8.1 | 4.9 ± 2.4 | 19.3 ± 9.2 | 17.4 ± 9.0 | 25.5 ± 9.5 | 15.3 ± 7.5 | 9.3 ± 5.4 | 87.1 ± 10.1 | 99.0 ± 6.5 |
| 45-49 | 122 | 81.4 ± 10.9 | 178.0 ± 5.9 | 25.7 ± 3.2 | 11.1 ± 4.5 | 15.6 ± 6.6 | 5.1 ± 3.1 | 16.9 ± 7.4 | 16.9 ± 7.9 | 25.8 ± 9.3 | 14.1 ± 6.6 | 8.7 ± 5.3 | 87.6 ± 9.2 | 98.0 ± 5.4 |
| 50-54 | 93 | 82.0 ± 11.5 | 177.1 ± 6.4 | 26.1 ± 3.2 | 11.7 ± 5.0 | 15.9 ± 5.8 | 5.1 ±2.6 | 17.9 ± 7.8 | 17.8 ± 8.3 | 28.0 ± 9.9 | 14.6 ± 6.8 | 8.4 ± 4.6 | 89.9 ± 10.6 | 99.1 ± 6.8 |
| 55-59 | 79 | 82.6 ± 11.3 | 176.6 ± 7.3 | 26.5 ± 3.2 | 13.7 ± 5.6 | 17.3 ± 6.4 | 5.8 ± 2.6 | 20.2 ± 7.3 | 19.3 ± 7.5 | 30.2 ± 6.5 | 16.4 ± 7.9 | 9.6 ± 4.6 | 92.4 ± 9.1 | 99.9 ± 6.1 |
| >60 | 94 | 81.1 ± 9.6 | 174.3 ± 7.3 | 26.7 ± 3.0 | 13.5 ± 4.9 | 17.9 ± 6.0 | 6.9 ± 3.5 | 19.4 ± 7.3 | 17.1 ± 6.4 | 27.4 ± 9.2 | 15.9 ± 8.0 | 8.9 ± 5.0 | 92.9 ± 9.6 | 100.3 ± 5.9 |
