# Supplementary material for: From fat to facts: Anthropometric references and centile curves for sum of skinfolds and waist-to-hip ratio in 2,507 adults
Source: PLoS One. 2025 Jun 26;20(6):e0326111. doi: 10.1371/journal.pone.0326111 (PMC12200776; doi:10.1371/journal.pone.0326111)
Supplement: S2 Table — (DOCX) [file pone.0326111.s002.docx]

| **Supplementary table 2**. Descriptive characteristics (mean ± standard deviation) of female participants according to age categories. | | | | | | | | | | | | | | |
| --- | --- | --- | --- | --- | --- | --- | --- | --- | --- | --- | --- | --- | --- | --- |
| Age | N. | Body mass (kg) | Height (cm) | BMI (kg/m^2^) | Triceps SKF (mm) | Subscapular SKF (mm) | Biceps SKF (mm) | Iliac crest SKF (mm) | Supraspinal SKF (mm) | Abdominal SKF (mm) | Tight SKF (mm) | Calf SKF (mm) | Waist girth (cm) | Hip girth (cm) |
| <20 | 56 | 65.8 ± 10.7 | 165.5 ± 8.6 | 24.0 ± 3.0 | 18.6 ± 7.1 | 15.0 ± 7.7 | 6.9 ± 4.3 | 17.0 ± 7.2 | 15.8 ± 7.8 | 19.4 ± 8.1 | 24.4 ± 10.4 | 14.0 ± 6.4 | 72.8 ± 7.0 | 98.7 ± 7.9 |
| 20-24 | 161 | 62.1 ± 9.8 | 164.1 ± 6.6 | 23.0 ± 3.1 | 19.7 ± 6.9 | 14.7 ± 7.6 | 7.5 ± 4.0 | 16.1 ± 7.7 | 14.9 ± 8.0 | 18.6 ± 8.1 | 27.5 ± 9.0 | 16.7 ± 7.7 | 70.0 ± 6.5 | 98.2 ± 7.4 |
| 25-29 | 155 | 62.8 ± 10.6 | 164.9 ± 7.1 | 23.0 ± 3.0 | 17.3 ± 6.7 | 15.0 ± 7.0 | 6.3 ± 3.1 | 15.8 ± 7.9 | 15.3 ± 7.5 | 19.0 ± 8.1 | 24.9 ± 8.7 | 15.6 ± 7.7 | 71.6 ± 7.8 | 97.7 ± 7.0 |
| 30-34 | 138 | 62.5 ± 10.2 | 164.7 ± 6.1 | 23.0 ± 3.2 | 18.5 ± 7.0 | 14.5 ± 6.8 | 6.7 ± 4.1 | 15.2 ± 8.4 | 15.3 ± 8.3 | 19.0 ± 9.0 | 26.5 ± 8.5 | 15.1 ± 7.4 | 71.0 ± 7.3 | 97.6 ± 7.7 |
| 35-39 | 122 | 62.1 ± 10.7 | 164.9 ± 6.0 | 22.8 ± 3.3 | 17.9 ± 6.5 | 14.5 ± 6.1 | 7.3 ± 3.6 | 15.2 ± 7.3 | 15.6 ± 7.5 | 19.7 ± 8.1 | 24.3 ± 7.8 | 14.9 ± 6.6 | 73.0 ± 8.3 | 97.4 ± 7.7 |
| 40-44 | 132 | 62.5 ± 10.1 | 163.6 ± 6.1 | 23.3 ± 3.2 | 19.1 ± 7.3 | 16.4 ± 7.1 | 7.5 ± 3.9 | 16.9 ± 8.0 | 16.4 ± 8.2 | 21.4 ± 8.6 | 26.4 ± 8.7 | 17.2 ± 7.4 | 74.8 ± 8.7 | 97.9 ± 7.5 |
| 45-49 | 117 | 65.1 ± 9.8 | 164.8 ± 6.1 | 24.0 ± 3.4 | 21.5 ± 7.6 | 18.7 ± 7.9 | 9.1 ± 4.7 | 19.4 ± 8.8 | 19.0 ± 8.6 | 24.0 ± 9.1 | 28.7 ± 9.6 | 19.3 ± 8.3 | 76.1 ± 9.7 | 98.7 ± 9.8 |
| 50-54 | 119 | 64.2 ± 8.7 | 162.5 ± 5.3 | 24.3 ± 3.3 | 21.9 ± 6.4 | 18.8 ± 7.5 | 9.4 ± 4.6 | 19.9 ± 9.0 | 20.1 ± 8.4 | 25.0 ± 8.5 | 28.5 ± 9.3 | 16.7 ± 6.7 | 77.9 ± 8.9 | 99.6 ± 6.9 |
| 55-59 | 103 | 65.3 ± 10.0 | 162.1 ± 6.2 | 24.9 ± 3.9 | 21.0 ± 7.4 | 19.0 ± 8.0 | 9.9 ± 4.7 | 19.9 ± 9.2 | 19.4 ± 9.0 | 24.9 ± 9.9 | 27.6 ± 9.6 | 16.8 ± 7.7 | 79.6 ± 10.2 | 99.8 ± 7.8 |
| >60 | 91 | 68.3 ± 10.9 | 161.6 ± 7.1 | 26.2 ± 4.4 | 20.7 ± 8.3 | 19.0 ± 7.3 | 10.0 ± 4.9 | 20.8 ± 7.9 | 18.8 ± 7.0 | 25.4 ± 10.7 | 25.0 ± 9.1 | 15.9 ± 8.2 | 85.7 ± 12.4 | 102.9 ± 8.7 |
