# Supplementary material for: From fat to facts: Anthropometric references and centile curves for sum of skinfolds and waist-to-hip ratio in 2,507 adults
Source: PLoS One. 2025 Jun 26;20(6):e0326111. doi: 10.1371/journal.pone.0326111 (PMC12200776; doi:10.1371/journal.pone.0326111)
Supplement: S3 Table — (DOCX) [file pone.0326111.s003.docx]

| **Supplementary Table 3.**  Sum of six skinfolds (mm) reference percentiles. | | | | | | | | | | | | | | |
| --- | --- | --- | --- | --- | --- | --- | --- | --- | --- | --- | --- | --- | --- | --- |
| Age (years) | 3rd | | 10th | | 25th | | 50th | | 75th | | 90th | | 97th | |
|  | Men | Women | Men | Women | Men | Women | Men | Women | Men | Women | Men | Women | Men | Women |
| <20 | 33.2 | 59.8 | 39.7 | 68.3 | 47.6 | 85.0 | 67.0 | 111.0 | 96.0 | 137.7 | 119.7 | 163.8 | 145.9 | 189.7 |
| 20-24 | 36.0 | 54.6 | 44.2 | 66.7 | 53.4 | 80.5 | 68.3 | 102.4 | 92.0 | 129.8 | 126.0 | 151.3 | 147.9 | 171.8 |
| 25-29 | 41.1 | 61.6 | 48.3 | 71.0 | 61.9 | 82.0 | 80.4 | 99.6 | 112.8 | 128.0 | 130.1 | 162.4 | 150.6 | 191.2 |
| 30-34 | 40.1 | 60.1 | 46.9 | 70.4 | 60.5 | 83.5 | 81.3 | 104.1 | 111.1 | 127.6 | 134.1 | 154.3 | 158.2 | 170.2 |
| 35-39 | 45.0 | 60.8 | 55.3 | 71.7 | 69.3 | 88.6 | 92.5 | 113.2 | 116.5 | 139.3 | 137.0 | 171.6 | 159.6 | 187.5 |
| 40-44 | 47.0 | 69.6 | 55.0 | 81.3 | 67.1 | 98.7 | 90.0 | 128.9 | 113.3 | 162.6 | 128.3 | 180.4 | 153.2 | 205.9 |
| 45-49 | 50.1 | 67.6 | 61.2 | 83.0 | 75.9 | 113.4 | 99.0 | 134.2 | 116.0 | 151.3 | 130.7 | 174.7 | 144.4 | 189.3 |
| 50-54 | 66.7 | 63.4 | 77.0 | 80.9 | 93.9 | 102.9 | 104.9 | 127.3 | 122.3 | 151.8 | 138.0 | 175.0 | 150.4 | 204.4 |
| 55-59 | 38.7 | 55.2 | 43.6 | 69.9 | 52.6 | 80.6 | 74.3 | 101.5 | 112.6 | 123.3 | 145.0 | 162.7 | 163.5 | 192.0 |
| ≥60 | 59.8 | 78.5 | 69.0 | 88.5 | 80.1 | 98.9 | 99.3 | 117.2 | 114.5 | 147.6 | 133.5 | 173.9 | 152.8 | 185.7 |
