# Supplementary material for: From fat to facts: Anthropometric references and centile curves for sum of skinfolds and waist-to-hip ratio in 2,507 adults
Source: PLoS One. 2025 Jun 26;20(6):e0326111. doi: 10.1371/journal.pone.0326111 (PMC12200776; doi:10.1371/journal.pone.0326111)
Supplement: S4 Table — (DOCX) [file pone.0326111.s004.docx]

| **Supplementary Table 4.** Waist-to-height ratio reference percentiles. | | | | | | | | | | | | | | |
| --- | --- | --- | --- | --- | --- | --- | --- | --- | --- | --- | --- | --- | --- | --- |
| Age (years) | 3rd | | 10th | | 25th | | 50th | | 75th | | 90th | | 97th | |
|  | Men | Women | Men | Women | Men | Women | Men | Women | Men | Women | Men | Women | Men | Women |
| <20 | 0.39 | 0.37 | 0.41 | 0.38 | 0.43 | 0.40 | 0.45 | 0.42 | 0.47 | 0.46 | 0.50 | 0.49 | 0.54 | 0.51 |
| 20-24 | 0.40 | 0.37 | 0.41 | 0.38 | 0.43 | 0.40 | 0.45 | 0.43 | 0.48 | 0.47 | 0.51 | 0.51 | 0.57 | 0.55 |
| 25-29 | 0.40 | 0.37 | 0.42 | 0.38 | 0.44 | 0.40 | 0.47 | 0.42 | 0.50 | 0.46 | 0.53 | 0.51 | 0.56 | 0.55 |
| 30-34 | 0.41 | 0.38 | 0.42 | 0.39 | 0.44 | 0.41 | 0.46 | 0.44 | 0.50 | 0.47 | 0.53 | 0.51 | 0.57 | 0.56 |
| 35-39 | 0.41 | 0.37 | 0.43 | 0.40 | 0.45 | 0.42 | 0.48 | 0.45 | 0.53 | 0.50 | 0.55 | 0.53 | 0.61 | 0.56 |
| 40-44 | 0.42 | 0.40 | 0.44 | 0.41 | 0.46 | 0.43 | 0.48 | 0.45 | 0.52 | 0.49 | 0.57 | 0.54 | 0.60 | 0.58 |
| 45-49 | 0.42 | 0.39 | 0.45 | 0.41 | 0.47 | 0.44 | 0.51 | 0.47 | 0.55 | 0.51 | 0.58 | 0.55 | 0.61 | 0.59 |
| 50-54 | 0.44 | 0.40 | 0.47 | 0.41 | 0.49 | 0.44 | 0.53 | 0.48 | 0.55 | 0.52 | 0.59 | 0.56 | 0.62 | 0.60 |
| 55-59 | 0.40 | 0.37 | 0.41 | 0.39 | 0.42 | 0.40 | 0.44 | 0.43 | 0.46 | 0.46 | 0.50 | 0.50 | 0.52 | 0.54 |
| ≥60 | 0.45 | 0.40 | 0.47 | 0.42 | 0.49 | 0.45 | 0.54 | 0.52 | 0.58 | 0.58 | 0.60 | 0.60 | 0.64 | 0.62 |
