# Supplementary material for: From fat to facts: Anthropometric references and centile curves for sum of skinfolds and waist-to-hip ratio in 2,507 adults
Source: PLoS One. 2025 Jun 26;20(6):e0326111. doi: 10.1371/journal.pone.0326111 (PMC12200776; doi:10.1371/journal.pone.0326111)
Supplement: S6 File — (PDF) [file pone.0326111.s006.pdf]

STROBE Statement—checklist of items that should be included in reports of observational studies

	Item No	Recommendation
**Title and abstract**	1	(*a*) Indicate the study's design with a commonly used term in the title or the abstract<br>Line 32: The present investigation was conceived as a multicenter, cross-sectional study.
		(*b*) Provide in the abstract an informative and balanced summary of what was done and what was found:<br>done
**Introduction**		
Background/rationale	2	Explain the scientific background and rationale for the investigation being reported<br>Lines 68-77: The quantification of body fat through anthropometric-based procedures derives mainly from the skinfold thickness measured in different body sites, inserted in predictive equations [6]. Although easier and cheaper than the laboratory techniques mentioned above, the measurement of the skinfold thickness requires accurate and reliable protocols defined by international guidelines [8]. Importantly, multiple predictive equations have been developed depending on factors like sex, age, and ancestry and are specific for athletes and non-athletes [6]. Consequently, this implies that the predictive equation for a given person should be chosen considering all factors together, and practitioners may not be aware of the existence of all the equations. Additionally, each equation has been developed using a specific population, and not being in line with the exact characteristics of such a population may result in a systematic error.
Objectives	3	State specific objectives, including any prespecified hypotheses<br>Line 93-94: the present study aimed to: i) provide centile references for Σ8SKF and WHR in adults and ii) provide lifespan analysis to estimate how both change over time.
**Methods**		
Study design	4	Present key elements of study design early in the paper<br>Line 113: The present investigation was conceived as a multicenter, cross-sectional study.
Setting	5	Describe the setting, locations, and relevant dates, including periods of recruitment, exposure, follow-up, and data collection<br>Lines 102 – 103: Recruitment took place through advertisements placed in universities, medical, recreational, market, and sports centers across Italy, starting in January 2024.<br>Lines 113-115: . The study collected data at a national level from multiple cities across various Italian territories (Milano, Padova, Reggio Emilia, Firenze, Chieti, Pescara, and Roma).
Participants	6	*Cross-sectional study*—Give the eligibility criteria, and the sources and methods of selection of participants
		Lines 106-109: The selection was also based on ensuring that at least 90% of the participants displayed a similar ethnicity. Exclusion criteria included the inability to collect all selected anthropometric measurements, pregnancy, or being outside the age

		range of 18 to 65 years.
Variables	7	Clearly define all outcomes, exposures, predictors, potential confounders, and effect modifiers. Give diagnostic criteria, if applicable<br>Line 115-123: The anthropometric assessments were conducted by operators certified by the International Society for the Advancement of Kinanthropometry, following international standards [8]. Body mass and stature were measured using a scale with an integrated stadiometer (Seca, Hamburg, Germany), with a sensitivity of 0.1 kg and 0.1 cm, respectively. BMI was calculated as body mass (kg) divided by squared stature (m^2).<br>Skinfold thicknesses at the triceps, biceps, subscapular, iliac crest, supraspinal, abdominal, thigh, and calf sites were measured using three different calipers (Holtain Ltd, United Kingdom; Harpenden, Baty International Ltd, West Sussex, UK; Cescorf, Porto Alegre, Brazil), each with a sensitivity of ±0.1 mm.
Data sources/ measurement	8*	For each variable of interest, give sources of data and details of methods of assessment (measurement). Describe comparability of assessment methods if there is more than one group<br>Line 115-123: The anthropometric assessments were conducted by operators certified by the International Society for the Advancement of Kinanthropometry, following international standards [8]. Body mass and stature were measured using a scale with an integrated stadiometer (Seca, Hamburg, Germany), with a sensitivity of 0.1 kg and 0.1 cm, respectively. BMI was calculated as body mass (kg) divided by squared stature (m^2).<br>Skinfold thicknesses at the triceps, biceps, subscapular, iliac crest, supraspinal, abdominal, thigh, and calf sites were measured using three different calipers (Holtain Ltd, United Kingdom; Harpenden, Baty International Ltd, West Sussex, UK; Cescorf, Porto Alegre, Brazil), each with a sensitivity of ±0.1 mm.
Bias	9	Describe any efforts to address potential sources of bias<br>Lines 103-107: We used a stratified sampling method based on age categories, within which individuals were randomly selected to ensure accurate representation. For each age group, a minimum number of subjects was recruited to ensure a normal distribution of the data, in accordance with previous studies [18,19]. The selection was also based on ensuring that at least 90% of the participants displayed a similar ethnicity.
Study size	10	Explain how the study size was arrived at 1,313 men and 1,194 women<br>Lines 105-106: For each age group, a minimum number of subjects was recruited to ensure a normal distribution of the data, in accordance with previous studies [18,19]
Quantitative variables	11	Explain how quantitative variables were handled in the analyses. If applicable, describe which groupings were chosen and why<br>Lines 152-153: Statistical analysis was conducted using R (version 3.4.1) and Lambda Mu and Sigma (LMS) method (LMS chart-maker Pro version 2.4, 2008). The mean ± standard deviation was calculated for each variable. Normal distribution of data was evaluated using the Shapiro–Wilk test. Smoothed age and sex-specific percentiles (3rd, 10th, 25th, 50th, 75th, 90th, and 97th) for Σ8SKF and waist-to-hip ratio were generated. The LMS method was used to graphically provide the annual rate of change of Σ8SKF and WHR, with three reference curves representing the median (M), the coefficient of variation (S), and the power to remove skewness from the data (L) by age and was implemented in the Generalized Additive Model for Location, Scale, and Shape (GAMLSS) package included in R software. In the LMS method,

GAMLSS parameters and the parameters of Box–Cox power exponential distribution were used for model fitting to data. These reference curves were fitted to the original data and the best fit was used to construct smoothed percentile curves. After the application of the BoxCox power transformation, the data at each age were normally distributed and the points on each percentile curve were defined in terms of the formula: M = (1 + LSz) 1/L where L, M, and S are values of the fitted curves at each age, and z indicates the z-score for the required percentile. For both sexes, simple linear regressions of the dependent variables (Σ8SKF and WHR) vs. the explanatory variable (age) were empirically investigated and tested for changes in the response variables' slope (Davies test) and for the existence of time points (Pscore test). To identify the time point(s) where a change in the slope of phase angle is observed, we performed a segmented regression analysis using the "segmented" package (v 1.0.0), selecting the model with the lower Bayesian information criterion value. Delta method and sandwich estimator for the standard errors were used to compute 95% confidence interval (CI) of the time point estimates. The slope coefficient estimates and the related 95% Cis were reported, and significant slopes were detected using p-value set at <0.05.

Statistical methods	12	(*a*) Describe all statistical methods, including those used to control for confounding
		(*b*) Describe any methods used to examine subgroups and interactions
		(*c*) Explain how missing data were addressed

Cross-sectional study—If applicable, describe analytical methods taking account of sampling strategy

Statistical analysis was conducted using R (version 3.4.1) and Lambda Mu and Sigma (LMS) method (LMS chart-maker Pro version 2.4, 2008). The mean ± standard deviation was calculated for each variable. Normal distribution of data was evaluated using the Shapiro–Wilk test. Smoothed age and sex-specific percentiles (3rd, 10th, 25th, 50th, 75th, 90th, and 97th) for Σ8SKF and waist-to-hip ratio were generated. The LMS method was used to graphically provide the annual rate of change of Σ8SKF and WHR, with three reference curves representing the median (M), the coefficient of variation (S), and the power to remove skewness from the data (L) by age and was implemented in the Generalized Additive Model for Location, Scale, and Shape (GAMLSS) package included in R software. In the LMS method, GAMLSS parameters and the parameters of Box–Cox power exponential distribution were used for model fitting to data. These reference curves were fitted to the original data and the best fit was used to construct smoothed percentile curves. After the application of the BoxCox power transformation, the data at each age were normally distributed and the points on each percentile curve were defined in terms of the formula: M = (1 + LSz) 1/L where L, M, and S are values of the fitted curves at each age, and z indicates the z-score for the required percentile. For both sexes, simple linear regressions of the dependent variables (Σ8SKF and WHR) vs. the explanatory variable (age) were empirically investigated and tested for changes in the response variables' slope (Davies test) and for the existence of time points (Pscore test). To identify the time point(s) where a change in the slope of phase angle is observed, we performed a segmented regression analysis using the "segmented" package (v 1.0.0), selecting the model with the lower Bayesian information criterion value. Delta method and sandwich estimator for the standard errors were used to compute 95% confidence interval (CI) of the time point estimates. The slope coefficient estimates and the related 95% Cis were reported, and significant slopes were detected using p-value set

	at <0.05.
	(*e*) Describe any sensitivity analyses

Continued on next page

**Results**		
Participants	13*	(a) Report numbers of individuals at each stage of study—eg numbers potentially eligible, examined for eligibility, confirmed eligible, included in the study, completing follow-up, and analysed<br>Lines 109-111: A total of 2,507 participants aged from 18 to 65 years, 1,313 men and 1,194 women were involved in this study. Lines 155-156: The detailed anthropometric characteristics of the participants are reported in Supplementary Table 1 and Table 2, for men and women, respectively.
		(b) Give reasons for non-participation at each stage
		(c) Consider use of a flow diagram
Descriptive data	14*	(a) Give characteristics of study participants (eg demographic, clinical, social) and information on exposures and potential confounders<br>Lines 155-156: The detailed anthropometric characteristics of the participants are reported in Supplementary Table 1 and Table 2, for men and women, respectively.
		(b) Indicate number of participants with missing data for each variable of interest
		(c) *Cohort study*—Summarise follow-up time (eg, average and total amount)
Outcome data	15*	*Cohort study*—Report numbers of outcome events or summary measures over time
		Case-control study—Report numbers in each exposure category, or summary measures of exposure
		Cross-sectional study—Report numbers of outcome events or summary measures<br>Lines 155-156: The detailed anthropometric characteristics of the participants are reported in Supplementary Table 1 and Table 2, for men and women, respectively.
Main results	16	(*a*) Give unadjusted estimates and, if applicable, confounder-adjusted estimates and their precision (eg, 95% confidence interval). Make clear which confounders were adjusted for and why they were included
		(*b*) Report category boundaries when continuous variables were categorized
		(*c*) If relevant, consider translating estimates of relative risk into absolute risk for a meaningful time period
Other analyses	17	Report other analyses done—eg analyses of subgroups and interactions, and sensitivity analyses
**Discussion**		
Key results	18	Summarise key results with reference to study objectives<br>Lines 225-233: The present study aimed to provide percentile curves for raw anthropometric measures of Σ8SKF and WHR in adult men and women. A second objective was to describe the rate of change of these parameters over the years. Percentile curves at the 3rd, 10th, 25th, 50th, 75th, 90th, and 97th percentiles were generated, and breakpoints were identified. For men, Σ8SKF showed a yearly increment of 1 mm from ages 21 to 59, while for women an increment of 3.8 mm per year was observed between ages 38.5 and 47. Regarding WHR, a progressive increment was recorded in men until the age of 28.4 years, with an annual rate of 0.004, followed by a slower increment of 0.003 per year in the subsequent ages. In women, the increment began at the age of 20, with a rate of 0.003 per year up to 65 years. Such novel data may facilitate the reference of body fat and WHR in adults.
Limitations	19	Discuss limitations of the study, taking into account sources of potential bias or imprecision. Discuss both direction and magnitude of any potential bias<br>Lines 298-304: The present study aimed to provide percentile curves for raw anthropometric measures of Σ8SKF and WHR in adult men and women. A second objective was to describe

		the rate of change of these parameters over the years. Percentile curves at the 3rd, 10th, 25th, 50th, 75th, 90th, and 97th percentiles were generated, and breakpoints were identified. For men, Σ8SKF showed a yearly increment of 1 mm from ages 21 to 59, while for women an increment of 3.8 mm per year was observed between ages 38.5 and 47. Regarding WHR, a progressive increment was recorded in men until the age of 28.4 years, with an annual rate of 0.004, followed by a slower increment of 0.003 per year in the subsequent ages. In women, the increment began at the age of 20, with a rate of 0.003 per year up to 65 years. Such novel data may facilitate the reference of body fat and WHR in adults.
Interpretation	20	Give a cautious overall interpretation of results considering objectives, limitations, multiplicity of analyses, results from similar studies, and other relevant evidence<br>Lines 151-277: In men, a constant accumulation of subcutaneous adipose tissue starts at 20 to 59 years old, then showing a plateau. A similar pattern has been observed when the total body fat percentage was monitored across the lifespan of male subjects [19]. Interestingly, adult people were observed to show a decrement in total energy expenditure up to 20 years old, then a plateauing up to 60, and then a further decrement [26]. As for men, this would imply that the total body fat accumulation may derive from other factors, should the total energy expenditure remain similar between 20 and 60 years old. Indeed, a decline in the growth hormone (GH) blood activity, which peaks during childhood and adolescence, begins to occur after the completion of physical growth [27]. In healthy adults, the age-related decline in growth hormone levels is accompanied by a parallel decrease in serum insulin-like growth factor 1 (IGF-1) [28]. The reduction in serum IGF-1 levels suggests a down-regulation of the GH/IGF-1 axis that has been strongly associated with increased fat accumulation and reduced muscle mass [28]. Although the reduction in total energy expenditure visible after 60 years old, the plateau in body fat accumulation may derive from the decrease in energy intake as well as in appetite reported with aging [29]. This seems associated with a combination of hormonal such as the decline in testosterone and leptine activity and psychological factors such as reduced physical activity or social isolation and age-related health conditions or medication side effects [30]. Additionally, the aging process is linked to reduced sensory perception in taste and smell, which may still decrease the interest in food [29,30].<br><br>Women exhibited a narrower window of body fat accumulation ranging from 38.5 to 47 years old, then showing a plateau. Such a period of increment could be a consequence of the perimenopausal phase which begins in the late 30s to early 40s [31,32] and marks the transitional period beginning with the first clinical, biological, and endocrine signs of impending menopause [31,32]. Indeed, during perimenopause women often experience changes in body composition, including decrements in muscle mass and increased body fat [31,32]. In addition, body fat may also change due to psychological aspects, including the influence of various diets and habits related to physical activity, often associated with the changes in hormonal status [33].
Generalisability	21	Discuss the generalisability (external validity) of the study results<br>Lines 307-314: The present study showed that subcutaneous adipose tissue increments during specific periods in both adult men and women, together with a progressive constant redistribution of body fat towards the abdominal area. It is now possible to refer to age- and sex-specific 3rd, 10th, 25th, 50th, 75th, 90th, and 97th percentiles, considering that time points of increment in Σ8SKF occur from 21 years to 59 years in men, and from 38.5 to 47 years in women. Meanwhile, WHR gradually increments after the age of 20 in men and after 23 in women. The new percentile references and the identified time points of change in this study can provide valuable support to researchers and practitioners for monitoring body composition

		and selecting appropriate nutritional and training strategies aimed at improving it.
**Other information**		
Funding	22	Give the source of funding and the role of the funders for the present study and, if applicable, for the original study on which the present article is based

*Give information separately for cases and controls in case-control studies and, if applicable, for exposed and unexposed groups in cohort and cross-sectional studies.

Note: An Explanation and Elaboration article discusses each checklist item and gives methodological background and published examples of transparent reporting. The STROBE checklist is best used in conjunction with this article (freely available on the Web sites of PLoS Medicine at http://www.plosmedicine.org/, Annals of Internal Medicine at http://www.annals.org/, and Epidemiology at http://www.epidem.com/). Information on the STROBE Initiative is available at www.strobe-statement.org.
